# Molecular Recognition and Specific Interactions for Biosensing Applications

**DOI:** 10.3390/s8106605

**Published:** 2008-10-23

**Authors:** Dong Chung Kim, Dae Joon Kang

**Affiliations:** BK 21 Physics Research Division, Institute of Basic Science, SKKU Advanced Institute of Nanotechnology, Center for Nanotubes and Nanostructured Composites, Sungkyunkwan University, Suwon 440-746, Korea. E-Mail: kimdc@skku.edu

**Keywords:** Molecular recognition, specific interaction, biosensor, nanopatterning, nanomaterial

## Abstract

Molecular recognition and specific interactions are reliable and versatile routes for site-specific and well-oriented immobilization of functional biomolecules on surfaces. The control of surface properties via the molecular recognition and specific interactions at the nanoscale is a key element for the nanofabrication of biosensors with high sensitivity and specificity. This review intends to provide a comprehensive understanding of the molecular recognition- and specific interaction-mediated biosensor fabrication routes that leads to biosensors with well-ordered and controlled structures on both nanopatterned surfaces and nanomaterials. Herein self-assembly of the biomolecules via the molecular recognition and specific interactions on nanoscaled surfaces as well as nanofabrication techniques of the biomolecules for biosensor architecture are discussed. We also describe the detection of molecular recognition- and specific interaction-mediated molecular binding as well as advantages of nanoscale detection.

## 1. Introduction

Biosensors usually employ immobilized biomolecules such as enzymes, antibodies or nucleic acids for the detection of analytes by converting a biological response into an electrical or optical signal [1]. The preparation of functional surfaces for the immobilization of only the desired biomolecules is the first process in the fabrication of biosensors. The functional surfaces provide ideal platforms for the immobilization of the desired biomolecules which can be achieved by physical adsorption, including electrostatic and hydrophobic interaction, covalent bonding, and specific interactions such as biotin-avidin, antibody-antigen interaction and DNA hybridization [2]. Especially the immobilization of biomolecules by the molecular recognition and specific interactions on the surfaces can yield good orientation and stability of the immobilized biomolecules thus leading to high-functionality within the biosensor fabrication process [3, 4].

Current advances in the field of nanobiotechnology have enabled the fabrication of novel biosensors at the nanoscale. The well-controlled immobilization of functional biomolecules on nanopatterned surfaces or nanomaterials via molecular recognition and specific interactions can provide higher sensitivity and specificity in the fabrication of biosensors. Biomolecular nanopatterning has been achieved by molecular self-assembly via molecular recognition and specific interactions in combination with various lithographic techniques (e.g. electron beam lithography, soft lithography, and dip-pen lithography) and synthesis of nanomaterials such as nanoparticles, nanorods, nanotubes, and nanowires [5].

This review focuses on molecular recognition- and specific interaction-mediated immobilization of functional biomolecules, nanoscale patterning of biomolecules using molecular recognition and specific interactions, and detection of the molecular binding at the nanoscale. Also the future direction as well as current techniques for the fabrication of novel biosensors at the nanoscale via the molecular recognition and specific interactions are discussed.

## 2. Surface functionalization for immobilization of functional biomolecules

The design of advanced surfaces, which offer controlled interaction with biomolecules for the recognition of analytes, is one of the most important factors in the fabrication and manipulation of biosensors [6-8]. The functionalization of the sensing surface can be accomplished by attachment of organic molecules via physical adsorption, covalent bonding or biochemical interaction etc. For the immobilization of only the desired biomolecules on the biosensor surface for detecting the target molecule, unwanted interactions of nonspecific molecules with biosensor must be prevented. Therefore the surface engineering to control the interface between the surface and conjugated biomolecules is a key prerequisite for practical applications of biosensors [9, 10]. The progress of surface chemistry and physics has led to the design of more efficient and highly selective surfaces at the nanometer scale, thus developing novel biosensors with high sensitivity and selectivity.

### 2.1. Self-assembled monolayer (SAM) and surface functionalization

Organic molecules can be well adsorbed onto solid-state surfaces such as metals and metal oxides because the molecules lower the free energy of the interface between the surfaces and the ambient environment [11]. The surfaces can be functionalized by self-assembled monolayers (SAMs), which are an easy and versatile system for organic assemblies [12]. The self-assembly process is mediated through non-specific adsorption, electrostatic interactions, covalent bonding and specific interactions. The SAMs are spontaneously formed by the adsorption/binding of organic molecules from solution or the gas phase onto a solid-state surface. The SAMs typically have a thickness of 1 – 3 nm and form a nanometer-scale organic thin film [11, 13].

Love *et al.* [11] summarized the headgroups of organic molecules that can bind to specific metals, metal oxides, and semiconductors. For example, the surfaces of metals such as gold and silver easily react with organic thiol groups and spontaneously form SAMs [14-17]. The hydroxyl groups of metal oxide surfaces like silicon dioxide can react with alkyltrichlorosilane or alkyltriethoxysilane such as 3-aminopropyltriethoxysilane and form covalent siloxane bonds at the surface [13, 18]. Gold forms good SAMs and does not react with atmospheric O_2_ and most chemicals as an inert metal [11]. A SiO_2_ surface exhibits high chemical stability and is widely applied in microelectronics [19, 20]. The ordered SAM formation of alkanethiolates on gold and alkylsiloxanes on SiO_2_ can provide hydrophilic or hydrophobic surfaces for nonspecific adsorption of biomolecules [21-24].

Since the headgroups of organic molecules directly bind or adsorb to the surfaces to form the SAMs, the property of functionalized surfaces is dependent on functional tailgroups of the molecules forming SAMs (Table 1). For example, amine groups on functionalized surfaces have positive charge at neutral pH, so they can interact with negatively charged biomolecules via electrostatic interaction. Moreover the amine tailgroup of the functionalized surfaces can form covalent bonding through 1-ethyl-3-[3-dimethylaminopropyl]-carbodiimide (EDC)-mediated reaction with carboxyl groups, glutaraldehyde-mediated reaction with amine groups, and chemical reaction with *N*-hydroxy-succinimide (NHS)-modified biomolecules.

**Figure 1.**
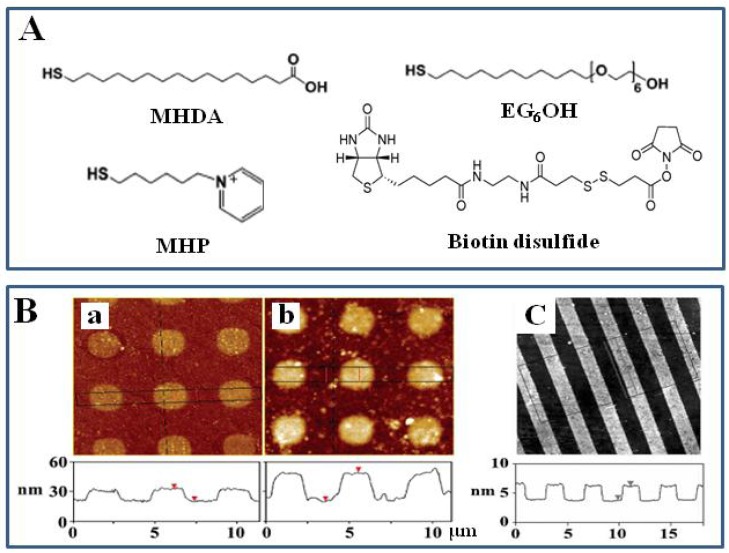
(A) Chemical structures of MHDA, MHP, EG_6_OH and biotin disulfide. (B) AFM topographic images showing the growth of 3D microscale structures. (a, b) A gold surface patterned by μCP with 2 μm dot arrays of MHDA and back-filled with MHP was used as the template to grow micropillars through the LBL assembly of PLL and PSS after 7 (a) and 12 (b) cycles. Reproduced with permission of ACS [25]. (C) AFM topographic image of μCP-patterned SAMs with 2 μm biotin-thiol stripes separated by EG_6_OH stripes on gold surface, Reproduced with permission of RSC [3].

We investigated surface engineering techniques using several SAMs (Figure 1A) such as 16-mercaptohexadecanoic acid (MHDA, a negatively charged carboxylic acid terminated thiol), 6-mercapto-*N*-hexylpyridinium bromide (MHP, a positively-charged pyridinium terminated thiol), 11-mercaptoundecyl hexa(ethylene glycol) alcohol (EG_6_OH, a neutral and flexible thiol terminated with hexa(ethylene glycol) group), and (2-[biotinamido]ethylamido)-3,3′-dithiodipropionic acid *N*-hydroxy-succinimide ester (biotin disulfide, a neutral and flexible disulfide group terminated with biotin) on gold substrate [3, 25]. Figure 1B shows the AFM topographic images of micropillars created by the LBL assembly of positively charged poly-l-lysine (PLL) and negatively charged poly(styrene-sulfonate) sodium salt (PSS) on microcontact printing (μCP)-patterned SAMs with 2 μm dot arrays of MHDA and backfilled with MHP on gold surface. PLL and PSS were sequentially self-assembled on 2 μm dots of MHDA and background of MHP via electrostatic interaction. Since the films of PLL/PSS assembled on the negatively charged MHDA surface grew much faster than those on the positively-charged MHP surface, the vertical difference between pillars and their background increased as a function of the cycle of LBL assembly using PLL/PSS. The differences in film growth characteristics can be applied in the fabrication of 3D micro-pillars simply by switching the template SAM coatings between the micro-dots and their background [25]. These charged surfaces can also be used as an effective platform for the immobilization of biomolecules via electrostatic interaction. Figure 1C shows the topographic image of μCP-patterned SAMs with 2 μm biotin-thiol stripes separated by EG_6_OH stripes on gold surface [3]. The EG_6_OH stripes are ∼2.4 nm taller than the biotin-thiol stripes. This agreed well with the previous data that the EG_6_OH and biotin-thiol SAMs are found to be ∼3.4 and ∼1.0 nm thick, respectively [26, 27]. A biotin layer can be an ideal platform for immobilization of biomolecules via biotin-avidin specific interaction while an EG_6_OH layer prevents the nonspecific adsorption of protein molecules [26, 28].

**Figure 2.**
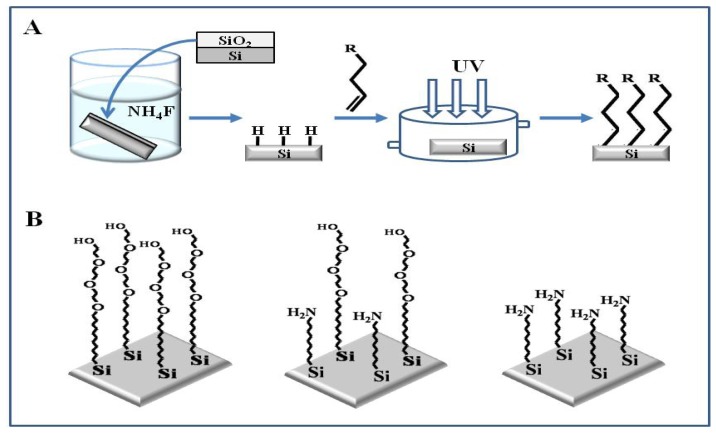
(A) Covalent modification of silicon. (B) The resulting monolayers of 100% EG3-ene, 50% Boc-*N*-ene + 50% EG3-ene, and 100% Boc-*N*-ene. Reproduced with permission of ACS [28].

As oligoethylene glycol monolayers like EG_6_OH have been used on Si/SiO_2_ surfaces as well as gold surfaces to resist the nonspecific adsorption of proteins and cells, these films may provide the best methods available to generate usefully inert surfaces [28-30]. The covalent modification of Si(111) surfaces through Si-C bond formation can provide a functional surface using direct photochemical reaction of vinyl groups with the surface that can serve as a stable surface for the immobilization of biomolecules [31, 32]. Figure 2 presents the fabrication of mixed monolayers showing both amine and ethylene glycol functionalities by applying solutions of various mole percentages of triethylene glycol undec-1-ene (EG3-ene) and *t*-Boc 10-aminodec-1-ene (Boc-*N*-ene) onto hydrogen-terminated silicon (111) surfaces. The surface functionalization using vinyl groups was achieved by UV illumination at 254 nm [28].

### 2.2. Layer-by-layer assembly

Layer-by-layer (LBL) assembly is a versatile and powerful bottom-up nanofabrication technique driven by electrostatic interaction, covalent bonding and specific interaction [33, 34]. As LBL assembly can create multilayer structures of biomolecules and be easily controlled, it has been used in the fabrication of biosensors with novel surface properties [34-36]. Although biomolecules can be LBL-assembled on surfaces using electrostatic interaction and covalent bonding [37-39], specific interaction via molecular recognition between host and target systems is a facile driving force for LBL assembly of biomolecules [10, 40]. LBL assembly has been carried out on patterned surfaces prepared by the top-down approach, including photolithography, soft lithography and dip-pen nanolithography, or on nanomaterials such as nanotubes, nanorods, nanowires and nanoparticles [7, 41].

## 3. Immobilization of functional biomolecules on functionalized surfaces

The most important criterion in the immobilization of functional biomolecules onto solid-state surfaces is to maintain the biological activity while retaining the 3D native structures [42-45]. The immobilization of functional biomolecules onto the functionalized surfaces has been achieved via physical adsorption including electrostatic and hydrophobic interaction, covalent bonding and specific interactions involving biotin-avidin, antibody-antigen interaction and DNA hybridization [2]. Table 2 shows the immobilization routes of biomolecules on functionalized surfaces. Each immobilization route has its own advantages and drawbacks with regard to stability, orientation, and functionality.

### 3.1. Physical adsorption

#### 3.1.1. Electrostatic interaction

The electrostatic interaction between charged molecules and oppositely charged surfaces has been shown to be quite an effective approach to immobilize biomolecules onto solid surfaces, especially in an immobilization process that requires no directional orientation of the biomolecules [46, 47]. The electrostatic interaction has been utilized to fabricate the optical and electrochemical biosensors via the immobilization of biomolecules onto solid-state surfaces [38, 48-50].

Recently polyelectrolytes have been used as a carrier to immobilize charged biomolecules [51, 52]. Myoglobin- and cytochrome-loaded films were fabricated using electrostatic interaction between charged biomolecules and oppositely charged films, respectively [52, 53]. These approaches using the electrostatic interaction between biomolecules and charged surfaces have provided a useful biomolecule-loaded film for fabricating novel biosensors, biocatalysts and biochips.

**Figure 3.**
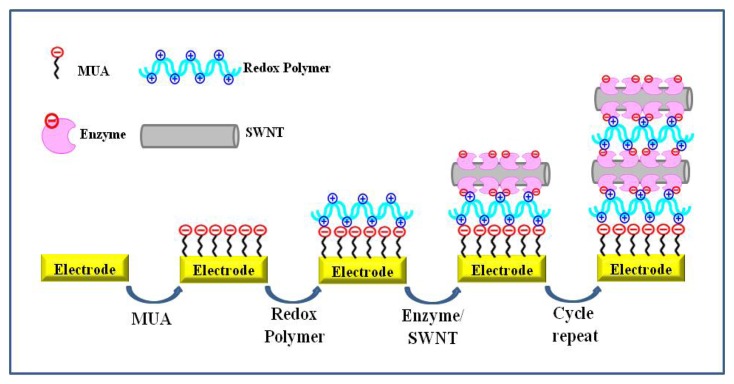
Schematic of the LBL film deposition process. Gold electrodes were first functionalized with the negative charged thiol, 11-mercaptoundecanoic acid (MUA), and then alternatively incubated in solutions of positively charged redox polymer and negatively charged enzyme/SWNT. Reproduced with permission of ACS [38].

Practically, a glucose biosensor [38] and a lactate biosensor [48] were fabricated via electrostatic LBL assembly. Electrostatic interaction-mediated immobilization of glucose oxidase (GOx) is shown in Figure 3. Gold electrodes were initially functionalized with negatively charged 11-mercapto-undecanoic acid followed by alternate immersion in solutions of a positively charged redox polymer, poly[(vinylpyridine)Os(bipyridyl)_2_Cl^2+^/^3+^], and a negatively charged GOx or GOx-modified single-walled carbon nanotubes (SWCNTs). Enzyme multilayer films with the desired number of layers can be achieved by repeating the electrostatic LBL assembly steps using positively charged polymer and negatively charged enzyme. The incorporation of SWCNTs resulted in an increase in the electrochemical response, and the sensitivity of the glucose sensors could be controlled by varying the number of multilayers [38]. Song *et al.* [54] demonstrated the fabrication of a hydrogen peroxide sensor using electrostatic interaction. Negatively charged DNA molecules were immobilized on the positively charged cysteamine-gold surface and then HRP molecules were assembled on the DNA-modified gold electrode. This biosensor showed high sensitivity, good reproducibility, and excellent long-term stability.

#### 3.1.2. Hydrophobic interaction

Hydrophobic interaction is a good alternative tool for the immobilization of biomolecules with a lipophilic property [55-57]. Catimel *et al.* [56] described the immobilization technique to investigate the interaction between immobilized ganglioside and its respective antibody for use in an optical biosensor. The ganglioside was immobilized by direct adsorption via hydrophobic interaction onto a gold-carboxymethyl dextran sensor chip without any derivatization of the sensor surface or the ganglioside. Lipophilic membrane-bound enzymes can be directly immobilized on hydrophobic surfaces for fabrication of biosensor. The membrane-bound enzymes such as D-fructose dehydrogenase (FDH), d-gluconate dehydrogenase and l-lactate dehydrogenase (LDH) were immobilized onto 5-(octyldithio)-2-nitrobenzoic acid (ODTNB)-functionalized gold electrodes by simple adsorption. Although the acid moiety of ODTNB may provide electrostatic interaction with the enzymes, the hydrophobic interaction between the largely lipophilic membrane-bound enzymes and the alkane moiety of ODTNB is the main force for adsorption [57]. The immobilized enzymes can detect the analytes by their catalytic activity.

### 3.2. Covalent bonding

In the immobilization of biomolecules on solid-state surfaces using the electrostatic interaction as a driving force, changes of environment such as ionic strength, pH or temperature can cause desorption of the adsorbed biomolecules [4, 58]. The covalent coupling route is more facile for the immobilization of biomolecules in terms of good stability and high binding strength [59]. Covalent Schiff-base bond [60, 61] and EDC-mediated attachment [62-64] have usually been used as suitable procedures for covalent bonding of biomolecules onto surfaces.

#### 3.2.1. Schiff-base reaction

The Schiff-base reaction between the aldehyde groups of biomolecules and amine groups of templates leads to the covalent attachment of biomolecules on solid-state surfaces [61]. Moreover the Schiff-base reaction using glutaraldehyde as a cross-linker provides a covalent bridge between amine groups of biomolecules and amine-functionalized surfaces under moderate conditions, which is highly useful for the construction of biosensors [65-67].

**Figure 4.**
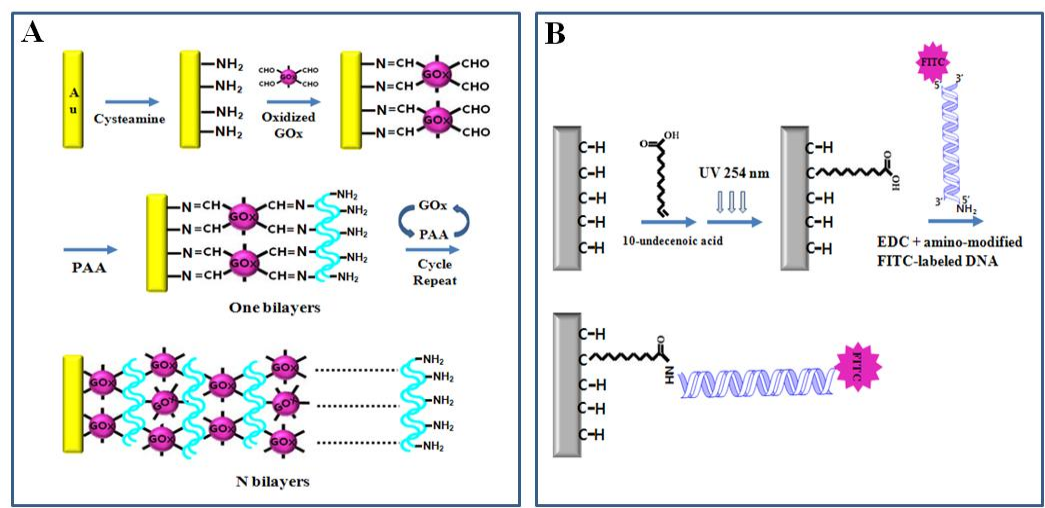
(A) LBL construction of GOx/PAA multilayer films via covalent bonding on the gold electrode surface. Reproduced with permission of Elsevier [61]. (B) Covalent attachment of FITC-labeled NH2-modified dsDNA to 10-undecenoic acid on NCD substrate using an EDC-mediated reaction. Reproduced with permission of Elsevier [62].

Schiff-base-mediated LBL assembly of GOx is shown in Figure 4A. A biosensor with good stability and reproducibility for glucose detection was fabricated by using LBL assembly of GOx/poly(allylamine hydrochloride) (PAA) multilayer films via covalent bonding. The cystamine was chemisorbed on the gold electrode and then GOx was covalently attached to the amine-modified electrode surface through reaction of aldehyde groups of the IO_4_^-^-oxidized GOx with amine groups of cysteamine adsorbed on the electrode surface. The PAA with polyamine groups was covalently attached to the aldehyde groups of the oxidized GOx on the electrode thus forming the first GOx/PAA bilayer. Covalently attached enzyme multilayer films with the desired number of layers can be prepared by repeating the LBL assembly steps [61].

#### 3.2.2. EDC-mediated and NHS-mediated chemical reaction

Water-soluble EDC-mediated reaction converts the carboxyl group into the unstable *O*-acylisourea intermediate that readily reacts with the amine group, resulting in covalent amide bonds between biomolecules and solid surfaces [63, 68]. Christiaens *et al.* [62] showed that amine-modified double-stranded DNA (dsDNA) was covalently attached to carboxy-modified diamond surface via EDC-mediated activation. 10-Decenoic acid was photochemically attached on the surface of nanocrystalline diamond (NCD) through irradiation with 254 nm UV light, and then fluorescein isothiocyanate (FITC)-labeled NH_2_-modified dsDNA was covalently attached to COOH-modified diamond using an EDC-mediated reaction (Figure 4B). The functionality of the covalently bound dsDNA molecules was confirmed by fluorescence measurements of FITC molecules. This covalent linking method for DNA immobilization can be applied to the DNA-based biosensor using complementary recognition of DNA.

Reactive NHS ester can also be directly reacted with an amine group to form a covalently stable amide bond [64, 68]. Covalent attachment of GOx to NHS-activated gold electrode led to improvement in stability and sensitivity in the fabrication of enzyme-based amperometric microbiosensor. Masson *et al.* [69] reported that GOx was spontaneously reacted with NHS ester of 16-mercaptohexandecanoic acid (NHS-MHA) on gold electrode. The self-assembled NHS-MHA covered approximately 93% of the electrode surface, thereby increasing the attachment sites for GOx and improving the diffusion of hydrogen peroxide to the gold electrode due to sufficient spacing of NHS-MHA layer for the enzymatic recognition.

### 3.3. Specific interaction

Although the non-specific immobilization of biomolecules via electrostatic interaction or covalent bonding on a solid-state surfaces may be the rapid, simple and cheap method, such non-specific immobilization of biomolecules on a solid-state surface often leads to highly random orientation of the immobilized biomolecules [70], and it may induce significant conformational change to the native 3D structure of biomolecules, thus causing severe loss of biological activity [42, 44]. To overcome these drawbacks of non-specific immobilization, biocompatible linkers between the surface and the biomolecule have been used [71-73]. The specific interaction-mediated immobilization of biomolecules using antibody-protein A/G interaction or DNA hybridization can prevent deterioration in functionality due to the improved orientation and stability [74, 76].

#### 3.3.1. Molecular recognition between biotin and avidin

The specific interaction based on molecular recognition of avidin-biotin provides a facile approach for the immobilization of biomolecules on solid-state surface. The avidin (or streptoavidin) based system is ideally suited for the well-controlled immobilization of biomolecules due to the specific and strong interaction (K_d_ ∼ 10^-15^ M) between avidin and biotin [73]. Since avidin has a nearly cubic shape with four biotin binding sites grouped in two pairs at opposite ends of the avidin molecule, it has been used for anchoring biotinylated biomolecules such as proteins and DNA thereby acting as a biocompatible linker between biotin and biotinylated biomolecules [10].

**Figure 5.**
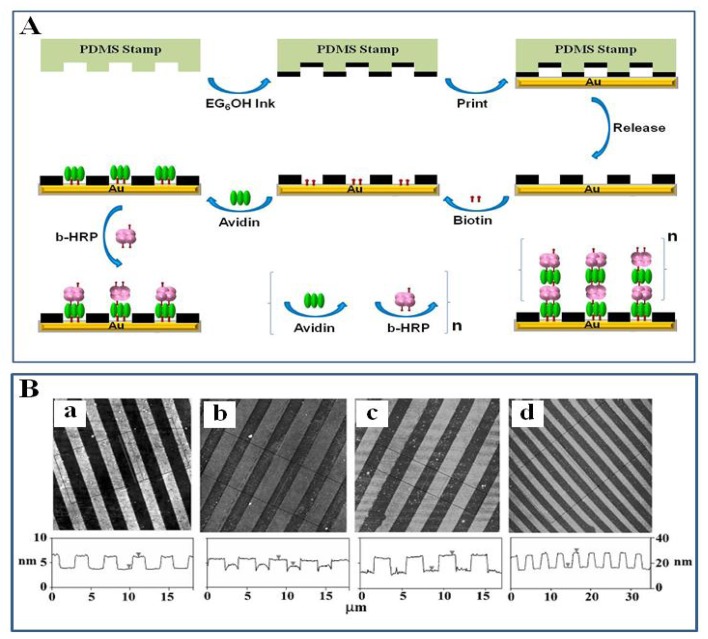
(A) Schematic of our approach to assemble 3D structures with active enzymes. (B) AFM topographic images showing the evolution of the avidin/biotin-HRP 3D structures. (a) A μCP-patterned surface; the EG_6_OH stripes are ∼2.4 nm taller than the biotin-thiol stripes. (b) After assembly of a bilayer of avidin/biotin-HRP, the protein stripes are ∼1.2 nm higher than the EG_6_OH stripes. (c) The same as **b** after a treatment with the surfactant; the protein stripes are ∼3.0 nm higher than the EG_6_OH stripes. (d) After assembly of 9 bilayers of avidin/biotin-HRP, the protein stripes are ∼12 nm higher than the EG_6_OH background [3].

We have shown that well-defined 3D nanostructures of the functional enzyme horse radish peroxidase (HRP) can be controllably fabricated on a μCP-patterned SAM template via a LBL assembly of avidin and biotin-HRP [3]. Figure 5 shows a schematic of our approach to build 3D nanostructures with active enzymes via biotin-avidin specific interaction. The gold surface was first patterned with micron-sized SAM template of EG_6_OH by μ-CP and then the micron-sized bare gold regions were functionalized with a biotin-terminated SAM. The EG_6_OH was used as a resist to prevent the non-specific adsorption of protein molecules [26, 28]. Avidin was then specifically assembled onto the biotinylated SAM regions via the bottom two of its biotin-binding sites. Biotin-HRP was built-up on the other two biotin-binding sites on the opposite side of the avidin. The activity of the immobilized biotin-HRP for hydrogen peroxide reduction was estimated to be ∼14% that of the free enzyme in solution. The level of catalytic activity is better than that of covalently immobilized HRP on flat surfaces representing ∼5% of free enzyme activity [75]. The enzyme multilayer films with the desired number of layers can be achieved by repeating the LBL assembly steps using avidin-biotin specific interaction. The level of catalytic activity sub-linearly increased as a function of enzyme layers, and the activity of 5- and 9-bilayer films increased ∼25% and ∼40%, respectively, compared to the first bilayer of avidin/biotin-HRP.

#### 3.3.2. Molecular recognition between protein A/G and antibody

In the fabrication of antibody-based biosensors, the site-specific immobilization of antibodies can strongly increase the antigen-sensing ability of immobilized antibodies on solid surfaces because it prevents the denaturation or conformational change of the antibody and also provides proper orientation of the antibody [74, 76]. Antibody-binding proteins such as protein A and protein G specifically bind the Fc region of an antibody, resulting in optimal antigen binding due to the oriented, functional antibodies which were bound to protein A/G without any modification [40, 45, 74]. The specific binding of the antibody leads to highly improved antigen detection compared to non-specific binding like covalent bonding or the physical adsorption of antibody [76-78]. Babacan *et al.* [45] compared the protein A-mediated specific binding with non-specifically glutaraldehyde-mediated covalent binding of antibody on a solid surface. The protein A-mediated immobilization of anti-*Salmonella* anitibodies was found to be better than the covalent immobilization due to the high stability and better reproducibility of the immobilized antibodies.

#### 3.3.3. Sequence-specific DNA hybridization

**Figure 6.**
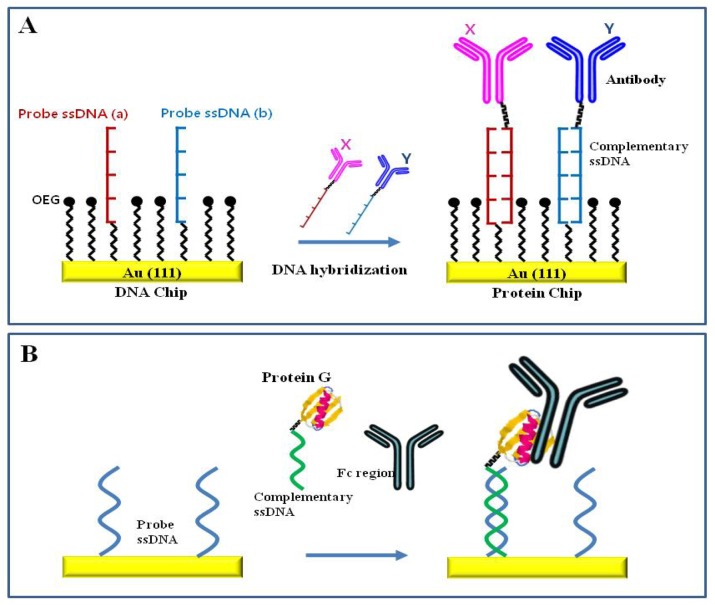
Schematic of (A) DNA-directed immobilization of DNA-protein conjugates using complementary hybridization of DNA. Reproduced with permission of ACS [72], and (B) DNA-directed antibody immobilization by the protein G-DNA conjugate. Reproduced with permission of ACS [40].

Site-directed immobilization of biomolecules via sequence-specific DNA hybridization is a stable and versatile method for the fabrication of biosensors [79-81]. DNA-directed immobilization of DNA-protein conjugates using complementary hybridization of DNA is shown in Figure 6A. An antibody-DNA conjugate was immobilized onto a patterned SAM of single stranded DNA (ssDNA) probe via sequence-specific DNA hybridization. Thiol-modified complementary ssDNA probes were attached onto a patterned gold surface via thiol-mediated chemical bonding. An oligo(ethylene glycol)-terminated thiols (OEG) background prevents the nonspecific binding of the DNA-protein conjugates to the surface. When a mixture of the conjugates was applied to the surface, the target conjugates self-assembled to the appropriate positions with complementary sequence via sequence-specific hybridization [72]. This surface with various ssDNA probes can be applied to the production of multichannel biosensors due to the diversity of complementary ssDNA conjugates, thus making it suitable in a DNA chip for the detection of target genes and in a protein chip for the detection of target antigens. The ssDNA chip can be converted into a protein chip via site specific hybridization of antibody-ssDNA conjugate and the protein surface can be converted back into a DNA surface by dehybridizing the complementary DNA [72, 79].

Also enzyme-DNA conjugates could be immobilized on a solid surface via site specific DNA hybridization. Fruk *et al.* [81] showed that a DNA-HRP conjugate on a microelectrode array retained substantial enzymatic activity for amperometric detection of hydrogen peroxide. The conjugate could be removed from the electrode surface by simple dehybridization and then fresh conjugate could be reloaded onto the electrode, thereby restoring the initial response activity.

DNA hybridization combined with biotin-avidin or antibody-protein A/G interactions can be a facile tool for immobilization of biomolecules. An immunoassay-based biosensor was fabricated by simultaneous use of biotin-avidin specific interaction and site specific DNA hybridization [80, 82]. Figure 6B shows an effective tool for the self-directed and self-oriented immobilization of antibody by protein G-DNA conjugate using both DNA-directed and protein G-mediated techniques. The specific immobilization of antibodies by protein G-DNA conjugate is well-controlled on the surface of biochips thus maintaining the activity and orientation of the bound antibody [40].

## 4. Nanoscale patterning of biomolecules using molecular recognition and specific interactions

Miniaturization of biosensors shall benefit from increase in the density of components, decrease in the amount and volume of a sample, improvement in detection limit, and precise control [83]. Recent advances in nanotechnology have enabled the development of miniaturized biosensors that are much smaller, more economical and more sensitive than current conventional biosensors. The ability to control surface properties on nanopatterned surfaces or nanomaterials is a key element for the nanofabrication of biosensors offering high sensitivity and specificity.

Nanofabrication of biosensor architectures can be achieved by either top-down or bottom-up approaches. The top-down approach for nanoscale patterning includes electron-beam lithography, soft lithography, nanoshaving, nanografting, dip-pen nanolithography, colloid lithography, block copolymer micelle lithography, and EUV-interference lithography [84, 85]. The bottom-up approach for nanofabrication of biosensors involves self-assembly of biomolecules onto nanomaterials by molecular recognition and specific interactions as well as physico-chemical synthesis of nanomaterials such as nanoparticles, nanorods, nanotubes and nanowires [86, 87]. High-resolution nanopatterning of biomolecules has been achieved by applying various kinds of lithographic techniques to self-assembled monolayers (SAM) containing LBL assembly [5]. By using the nanopatterning techniques, biomolecules can be positioned within desired nanoregions with well-defined feature size and shape while retaining their native 3D structures and biological functionalities [85].

A wholly comprehensive review of nanofabrication techniques is beyond the scope of this review. So we narrow our viewpoint to five well-known lithographic techniques- electron-beam lithography (EBL), nanocontact printing (NCP), nanoimprnit lithography (NIL), nanografting, nanoshaving, and dip-pen nanolithography (DPN). Furthermore this section focuses on the biomolecular nanopatterning using only molecular recognition and specific interactions combined with the six lithographic techniques. Table 3 presents current techniques for biomolecular nanopatterning as well as the advantages and limitations of these different lithographic techniques with regard to resolution, cost, speed, simplicity, and arbitrary patterning [84, 85, 88].

### 4.1. Electron-beam lithography (EBL)

**Figure 7.**
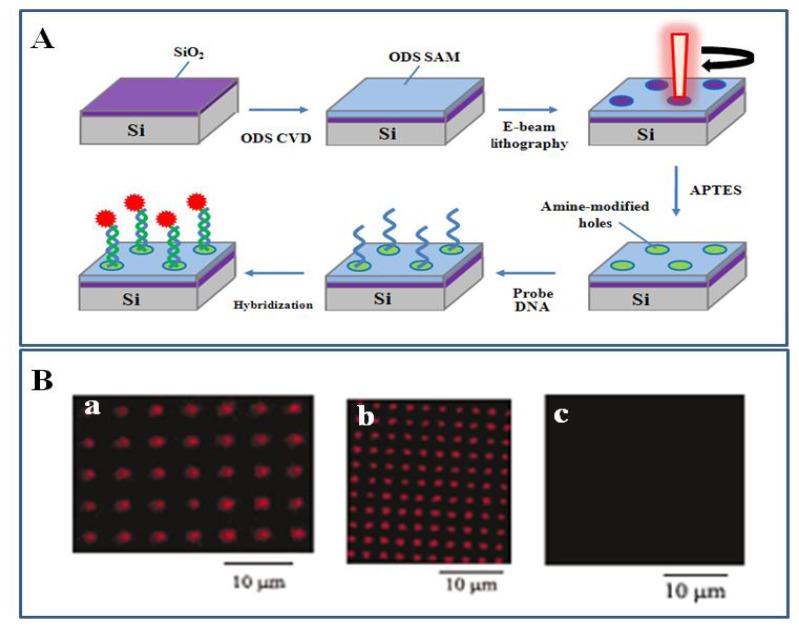
(A) Schematic of fabrication of nanometer-sized patterns using EBL. (B) Fluorescence micrographs of Cy 5 labeled target hybridized to probe DNA immobilized within nanodot areas fabricated by EBL on ODS SAM: (a) complementary probe DNA (spot size: 500 nm); (b) complementary probe DNA (spot size: 250 nm); (c) non-complementary probe DNA. Reproduced with permission of RSC [83].

The most general method of the top-down approach is photolithography, which is the process that creates micron-scaled patterns on flat solid surfaces by exposure to UV light through a mask. However, the use of photolithography is limited by the diffraction limit of UV light and the production of masks. EBL as a maskless lithography can create nanoscaled fine patterns on flat surfaces with a beam of electrons [84]. Nanopatterning of SAM combined with the EBL produces an organic template for various nanofabrication of biosensors [83, 94]. Biomolecules such as DNAs and proteins are immobilized on nano-patterned organic templates provided by EBL [95, 96]. DNA could be immobilized via sequence-specific DNA hybridization on EBL-nanopatterned surfaces. Nanometer-sized patterning of DNA using EBL is shown in Figure 7.

Octadecyltrimethoxysilane (ODS) monolayer for preventing non-specific adsorption of biomolecules was formed on SiO_2_ surfaces by chemical vapor deposition and then patterned by a beam of electrons on the ODS-functionalized SiO_2_ surfaces to regenerate the sianol groups. 3-Aminopropyl-triethoxysilane (APTES) was easily deposited in the empty regions of pin-hole shapes on the nanopatterned surface and then amine-modified oligonucleotides were covalently attached on nanopatterned APTES regions by glutaraldehyde-mediated chemical reaction. The hybridization between immobilized probe ssDNA and fluorescence-labeled complementary ssDNA showed strong fluorescence within the nano-patterns owing to DNA-DNA complementary interaction, while immobilized ssDNA can not interact with non-complementary ssDNA [83]. This exhibited the tool for creating well-defined DNA nanostructures on solid surfaces, thus applying to the development of biosensors via molecular recognition of complementary DNA.

Zhang *et al.* [94] also developed a tool for creating protein nanopatterns with high resolution using EBL in combination with a LBL assembly via high-affinity biotin-avidin interaction. A SAM of 1*H*,1*H*,2*H*,2*H*-perfluorodecyltriethoxysilane (FDTES) was deposited onto a silicon surface by chemical vapor deposition (CVD), and then nanoscale patterns of APTES SAMs were created by using EBL, followed by the covalent immobilization of biotin on the nanopatterned regions. Streptavidin was subsequently immobilized on nanopatterned regions of biotin, and finally a biotinylated green fluorescence protein (GFP) was bound with the streptavidin through biotin–avidin interactions. The nanopatterned proteins retained their biological activity. The fluorescence created by specific binding of biotinylated GFP to streptoavidin represented the high resolution and accuracy in the nanoscale pattern. The use of biotinylated enzymes or antibodies with specific function instead of biotinylated GFP enables the development of nanoscale biosensors via biotin-avidin specific interaction.

### 4.2. Soft lithography

As EBL is a very slow and expensive process usually requiring large investments such as clean room and high vacuum, it is restricted by the difficulty in efficient application for biosensor fabrication [84]. Soft lithography is a non-photolithographic method based on printing and replica molding using elastomeric stamps for micro and nanopatterning as well as self-assembly [14, 97]. The development of soft lithography, which is a process for creating fine patterns on flat surfaces by using elastomeric stamps or replica mold, enables scalable, parallel and cost effective nanopatterning [98]. The representative tools in soft lithography for nano-patterning are nano-contact printing (NCP) and nanoimprint lithography (NIL) [99].

#### 4.2.1. Nanocontact printing (NCP)

NCP does not require complicated and expensive facilities. NCP forms nanopatterns of SAM of inks on solid surfaces through direct contact with poly(dimethylsiloxane) (PDMS) stamps. PDMS stamps are created by curing liquid prepolymers of PDMS on an EB lithographically fabricated master [100]. The stamps are dipped in the ink with desired molecules and then the contact of inked stamp on the surface transfers the desired molecules from the stamp to the surface [88]. Since low mechanical stability of the stamp and diffusion of low molecular weight SAM inks during the printing step often limit the minimum feature size attainable, ultrasmall nanopatterns can be achieved via a combination of sharp and hard PDMS stamps and high molecular weight inks to avoid diffusion. Practically the decrease in feature size of the PDMS stamp and contact area as well as diluting the protein solution led to 100 nm-scaled patterns of immunoglobulin G (IgG) and GFP on glass surface [100]. This technique enabled biomolecular nanopatterning at a resolution of less than 50 nm [90].

#### 4.2.2. Nanoimprint lithography (NIL)

NIL employs the replica mold for nanopatterning, where the stamp is only used to create physical features in the polymer coated substrates. The solid surface is spin-coated by thermoplastic polymer film such as poly(methyl methacrylate) (PMMA) and then heated above the glass transition temperature of the polymer. The rigid mold is pushed into polymer film and after cooling below the glass transition temperature the mold is separated from the surface [101, 102]. The surface is patterned by sequential etching and self-assembly processes. The patterned surface created by NIL can be then functionalized to produce nanostructures of biomolecules. NIL was capable of selective patterning of bioactive proteins with nanoscale resolution of ∼75 nm [91].

Ohtake *et al.* [103] fabricated biomolecular nanopatterns for the detection of DNA hybridization using a NIL technique. Probe ssDNA was immobilized on PLL-coated glass substrate via electrostatic interaction and then UV exposure strengthened the bonds between the ssDNA and the PLL by producing radical species for each molecule. Polyvinyl alcohol (PVA) was spin-coated on the immobilized DNA layer. A DNA nanopattern was formed on PVA surface by nanoimprinter with a mold. The ssDNA nano-arrays can be used to detect a sequence-specific DNA hybridization.

**Figure 8.**
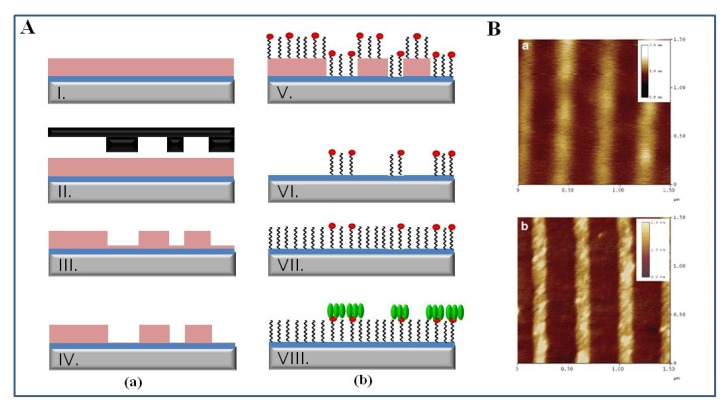
(A) Schematic of biomolecular nanopatterning using NIL in combination with molecular self-assembly. (B) AFM scans in tapping mode in air of 100 nm patterned stripes. (a) PLL-*g*-PEG/PEG-biotin stripes in an oxide background (after lift-off, stage VI in Figure 8A-b). (b) After PLL-*g*-PEG backfill the pattern is still visible due to the longer PEG chains supporting the biotin molecules (stage VII in Figure 8A-b). Reproduced with permission of ACS [104].

Protein nanopatterning with nanoscale resolution using NIL in combination with molecular assembly patterning by lift-off (MAPL) is shown in Figure 8. PMMA film on niobium oxide (Nb_2_O_5_) surface was imprinted by a silicon stamp. The PMMA/Nb_2_O_5_ surface produced by NIL and dry etching process was incubated into an aqueous solution of PLL-*graft*-poly(ethylene glycol)-biotin (PLL-*g*-PEG/PEG-biotin). The PLL-*g*-PEG/PEG-biotin was immobilized on Nb_2_O_5_ patches via electrostatic interaction between the positive charge of PLL-*g*-PEG/PEG-biotin and negative charge of the surface at neutral pH. After non-specific adsorbed PLL-*g*-PEG/PEG-biotin on PMMA was eliminated by PMMA lift-off, the empty background was filled with protein-resistant PLL-*g*-PEG. Streptavidin was selectively immobilized on the biotin areas via avidin-biotin specific interaction. The streptavidin patterns with 100 nm feature sizes can be used as a universal platform for the immobilization of biotin-tagged biomolecules such as biotin-enzyme, biotin-antibody and biotin-DNA, thus applying to development of biosensors and biochips [104].

### 4.3. Nanografting and Dip-pen nanolithography

Nanografting, nanoashaving and dip-pen nanolithography (DPN) are a direct-writing, scanning probe-based lithographic techniques providing high resolution. They can produce nanoscale patterns for the immobilization of biomolecules by using atomic force microscopy (AFM). The nanopatterns are created on a surface by mechanical scraping or from ink deposition using AFM tips [88, 105].

#### 4.3.1. Nanografting and Nanoshaving

In the nanografting technique, an AFM tip scrapes away patterns on an existing biomolecule-resistant SAM and then the grafted areas are filled with new biomolecule-adherent SAM. The biomolecules can be immobilized onto the grafted areas by using specific interaction via molecular recognition between immobilized biomolecules and the grafted areas with biomolecule-adherent SAM [106]. Nanografting was capable of biomolecular nanopatterning with nanoscale resolution of ∼10 nm. Nanopatterns of thiolated ssDNA were created on a gold surface by using nanografting, thus leading to sequence-specific DNA hybridization [92]. In the nanoshaving technique, the AFM tip mechanically removes a resister for creating nanopatterns on surfaces and then biomolecules can be immobilized onto the nanoshaved patterned surfaces using a sequential self-assembly [85, 107].

**Figure 9.**
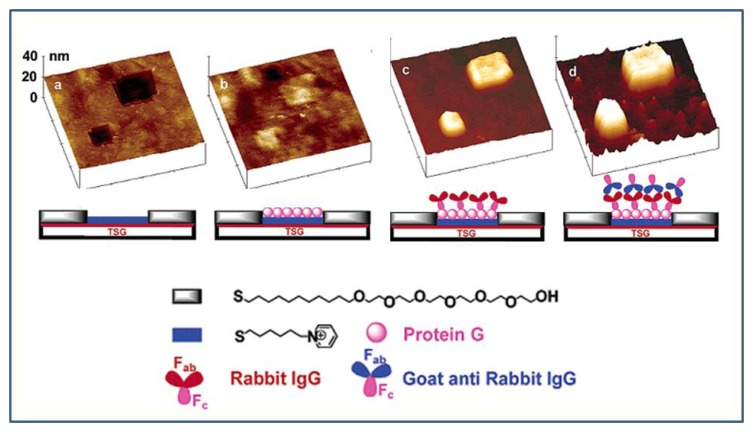
3D AFM topographic images showing the process of creating and growing the 3D protein nanostructures by electrostatic immobilization and biospecific interaction. (a) Two nanopatches (200 × 200 and 400 × 400 nm^2^) created by nanografting of MHP into the SAM of EG_6_OH had a depth of 2 nm. (b)After incubation with protein G, the patches were completely filled by protein G with an average height of ca. 1 nm. (c) After treatment with rabbit IgG, the height of the protein nanofeatures increased to 8-9 nm. (d) After subsequent treatment with goat anti-rabbit IgG, the height of the protein nanofeatures increased to 19-20 nm. Reproduced with permission of ACS [108].

Biomolecular nanopatterning using the nanografting technique is shown in Figure 9. The positively charged nanoholes were created by nanografting of MHP within a SAM resist of EG_6_OH. As protein G, an immunoglobulin-binding protein, is negatively charged in neutral pH, it was adsorbed onto positively charged MHP nanoholes via electrostatic interactions. The immobilized protein G onto nanostructured surfaces led to specific oriented attachment of IgG via antibody-protein G specific interaction, thus constructing novel antibody-based biosensors and biochips [108]. Interestingly antibody-functionalized nanotubes could be specifically immobilized onto a nanografting-patterned surface via antibody-antigen specific interaction. Trenches were shaved into a SAM of 1-octadecanethiol on gold surface using the AFM tip and then antigen was adsorbed on the trenches via thiol-gold interaction. The antibody-functionalized nanotubes were site-specifically assembled on the complementary antigen regions of the trenches via molecular recognition [109]. Furthermore, multiple DNA nanopatterns of two different sequences were produced on a single gold surface by nanoshaving technique and applied to selective label-free DNA detection [107].

#### 4.3.2. Dip-pen nanolithography (DPN)

DPN is a direct-writing technique for nanopatterning on solid surfaces using an ink-coated AFM tip. The molecules are directly transferred from the AFM tip to the solid surface via diffusion through a water meniscus under ambient conditions [105, 110]. DPN enables biologically active protein nanoarrays on various solid surfaces [111-112].

Lee *et al.* [111] demonstrated nanopatterning of antibody and lysozyme on gold surfaces by DPN. The negatively charged nanopatterns (100-300 nm) with a SAM of 16-mercaptohexadecanoic acid (MHA) were created by using an AFM tip and the background was then filled with a SAM of 11-mercaptoundecyl tri(ethylene glycol) to prevent non-specific adsorption of proteins. The IgG and lysozyme were non-specifically immobilized onto the MHA patterns where the proteins retained bioactivity. Anti-IgG specifically assembled on IgG nanopatterns via antibody-antigen specific interaction.

**Figure 10.**
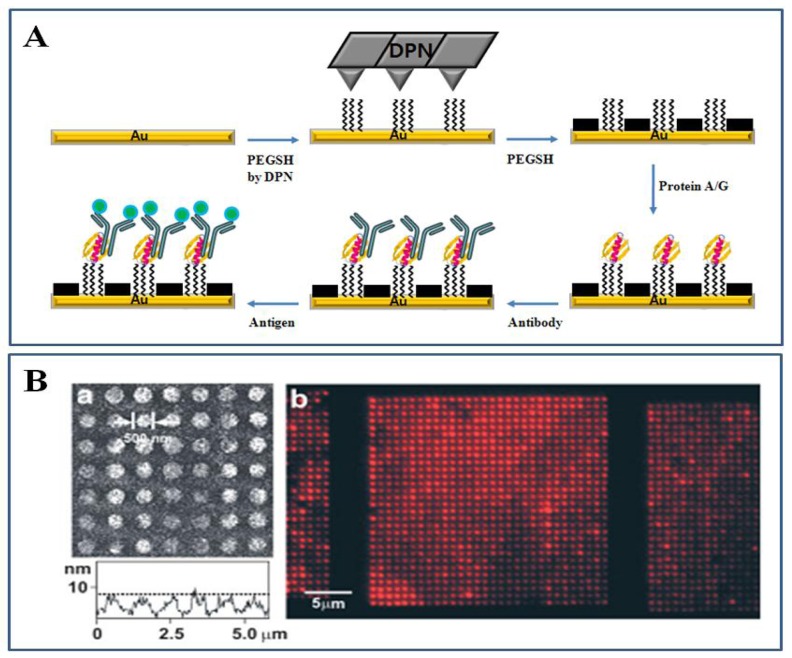
(A) Schematic representation of the process used to obtain biologically active antibodies on the protein A/G, which is covalently bound to NHSC_11_SH nanoscale features patterned using DPN. (B) IgG was immobilized on protein A/G nanoarrays. (a) Topographical TMAFM image and its corresponding height profile of fluorescein isothiocyanate Alexa Fluor 594-labeled human IgG nanoarrays immobilized onto protein A/G templates. (b) Representative fluorescence microscopy image of Alexa Fluor 594-labeled antibody nanoarray patterns. Reproduced with permission of Wiley [112].

Interestingly parallel DPN could generate multiple nanoarrays of biologically active proteins, as illustrated in Figure 10. Nanoarrays of a SAM of 11-mercaptoundecanoyl-*N*-hydroxysuccinimide (NHSC_11_SH) were created on gold surface by using parallel AFM tips and the background was then filled with a SAM of 11-mercaptoundecyl tri(ethylene glycol) (PEGSH). Protein A/G was covalently attached onto the DPN-generated NHSC_11_SH nanoarrays via covalent bonding between NHS groups of NHSC_11_SH and amine groups of proteins.

As protein A/G specifically binds the Fc region of an antibody and optimizes antibody orientation as well as minimizes antibody denaturation, the biologically active antibody was immobilized on the nanoarrays of protein A/G, resulting in application in antibody-based biosensors for detection of specific antigens [112]. Also DPN was capable of direct DNA nanopatterning on metals and insulators. Surfaces were functionalized at the nanoscale with the desired DNA by DPN, and then the complementary DNA was selectively hybridized to DPN-generated nanoscaled regions via molecular recognition [113, 114].

### 4.4. Molecular recognition and specific interaction of biomolecules on nanomaterials

Various nanomaterials have been fabricated using chemical synthesis, chemical vapor deposition (CVD), physical vapor deposition (PVD), and template-based electrodeposition. Nanomaterials such as nanorods, nanowires, nanotubes, and nanoparticles have potential applications in the fabrication of biosensors because of their unique electronic, optical, chemical, and mechanical properties [115]. The nanomaterals can be a potential platform in the immobilization of biomolecules using molecular self-assembly for the fabrication of biosensors due to their high surface area and high surface free energy [116]. As well as non-specific interactions such as electrostatic interaction or covalent bonding [117-119], specific interactions based on molecular recognition are reliable and versatile routes for immobilization of biomolecules on nanomaterials [120-122].

#### 4.4.1. Nanotubes

**Figure 11.**
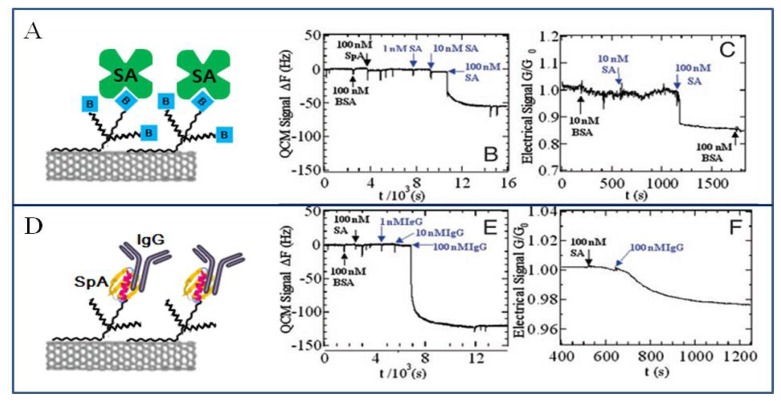
Real-time quartz crystal microbalance (QCM) and electronic sensing of specific biological recognition on nanotubes. (A) Scheme for streptavidin (SA) recognition with a nanotube coated with biotinylated Tween. (B) QCM signal; Biotin-nanotubes binds SA specifically but not other proteins. (C) Conductance; Specific binding of SA is detected electronically. (D) Scheme for IgG recognition with a nanotube coated with a SpA–Tween conjugate. (E) QCM signal; SpA-nanotubes binds IgG specifically but not unrelated proteins. Note that 10 nM IgG concentration approaches the lower detection limit of the instrument, whereas 100 nM approaches surface saturation of the sample; thus, the response does not show a full proportionality to the concentration. (F) Conductance; Specific binding of IgG is detected electronically. Reproduced with permission of APS [125].

The carbon nanotube (CNT) is a promising material for biosensing applications due to its high surface-to-volume ratio, its biocompatibility, and the fast electron-transfer mediation for wide range of electroactive species [123, 124]. Real-time sensing of specific biological recognition on nanotubes is shown in Figure 11. A SWCNT was noncovalently functionalized by using specific interaction via molecular recognition of biomolecules. The biotinylated Tween-20 was adsorbed onto the SWCNT, and then the SWCNT coated with biotinylated Tween-20 bound with streptavidin specifically but not with other proteins. The specific binding of streptavidin was detected electronically (Figure 11A). Besides the Staphylococcus protein A (SpA)-conjugated Tween-20 was adsorbed on SWCNT, and then the SWCNT coated with SpA-Tween conjugate bound with antibody specifically but not with other proteins (Figure 11D). The specific binding of antibody was detected electronically [125]. The specific binding of biotinylated enzymes with the streptavidin-conjugated SWCNT and that of specific antigen with the antibody-conjugated SWCNT can be applied in the fabrication of specific electronic biosensors using enzymatic reaction and using antigen-antibody molecular recognition, respectively.

O'Connor *et al.* [121] presented the fabrication of amperometric immune-biosensor on SWCNT forests. The biotin-antibody was adsorbed on SWCNT forests and then the biotin-antiboby-conjugated SWCNT bound with biotinylated HRP. The specific binding of biotinylated molecules was detected electronically. The biotinylated HRP immobilized on the biotin-antiboby-conjugated SWCNT can be used for detecting hydrogen peroxide.

Biosensors were fabricated on gold nanotubes using the molecular recognition of biotin-streptavidin and protein G-antibody. Streptavidin was bound on biotininylated gold nanotubes (biotin-Au NT), and biotinylated protein G was built-up on the streptavidin-biotin-Au NTs. Anti-ricin antibody was bound on the protein G-streptavidin-biotin-Au NT and then ricin as an antigen was finally bound on the antibody-protein G-streptavidin-biotin-Au NT. The sequential bindings of streptavidin, protein G, antibody and antigen could be electrochemically detected through biofunctionalized conical gold nanotubes [122].

#### 4.4.2. Nanorods and nanoparticles

**Figure 12.**
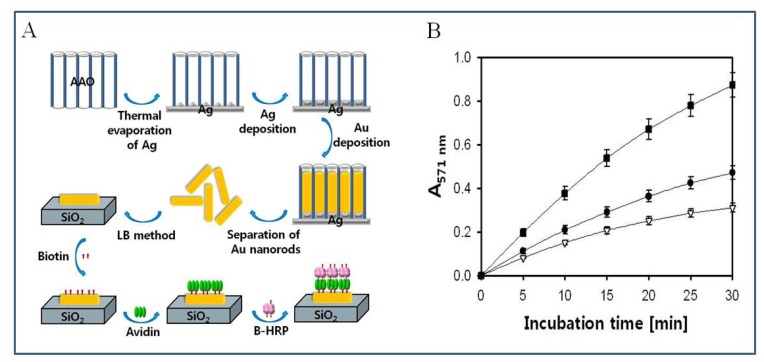
(A) Schematic diagram of preparation of the dispersed film of gold nanorods and fabrication of bio-architecture with HRP activity on gold nanorod. (B) The catalytic activity of LBL-assembled HRP on gold nanorods compared to that on a flat gold surface. Closed square, on vertical array of gold nanorods; Closed circle, on dispersed film of gold nanorods; Open triangle, on flat gold surface. Reproduced with permission of KPS [126].

Recently we described the fabrication of bio-architecture with enhanced catalytic activity on gold nanorods using surface templated LBL assembly via biotin-avidin specific interaction, illustrated in Figure 12A [126]. Gold nanorod structures were constructed through porous aluminum oxide as a template, and the bio-architecture was fabricated on the gold nanorods through LBL assembly for the colorimetric detection of hydrogen peroxide. The bio-architectures (0.5×0.5 cm^2^) were reacted with 5 mM hydrogen peroxide in a buffer solution with 50 μM Amplex red and their activities were measured by monitoring the peak absorption at 571 nm. The level of catalytic activity of the bio-architectures with avidin/biotin-HRP on both the vertical array and on the dispersed film of gold nanorods was found to be ∼ 2.8 and ∼1.5 times better than that of the flat gold surface, respectively, due to increased amounts of immobilized HRP (Figure 12B). This nanorod structure provides the increased surface area for enzyme immobilization in biosensors, thus leading to enhanced sensitivity [119].

Endo *et al.* [127] fabricated an optical biosensor based on localized surface plasmon resonance on surface modified gold-capped nanoparticle layer. The anti-fibrinogen antibody was immobilized onto the surface of nanoparticle layer via protein A-antibody specific interaction. The detection of specific binding of fibrinogen to the anti-fibrinogen antibody was measured by the change in the absorption spectrum, caused by the antigen-antibody reaction and the concentration of antigen. Xiao *et al.* [128] showed the immobilization of redox protein, GOx, on gold nanoparticles using cofactor-apoenzyme specific interaction. The amino derivative of the FAD cofactor was covalently attached to the NHS-functionalized gold nanoparticles which were linked to a gold electrode surface by a dithiol monolayer. The GOx was reconstructed by specific binding of apo-GOx on the FAD-functionalized gold nanoparticles via molecular recognition. This system led to effective electron transfer from the FAD moiety to the electrode, thus yielding a glucose biosensor with high sensitivity and selectivity [128, 129].

## 5. Nanoscale detection of molecular recognition and specific interactions

### 5.1. Advantages of nanoscale detection

Advances in nanotechnology could provide great advantages in the development of ultra-small biosensors with improved sensitivity. Nanopatterning of biomolecules could be achieved by using top-down approaches in combination with bottom-up approaches. Nanopatterning techniques enable nanoarrays with smaller and more densely packed features. The biomolecular nanoarray benefits from smaller biosensors with more reaction/binding sites, higher sensitivity, and smaller sample volumes due to an increase in the density of receptor elements and in a decrease in the array size [112, 130, 131]. Biomolecular nanoarrays with ultra-small features may become a powerful tool for studying single-biomolecules such as DNA, proteins and viruses, thereby allowing for the discovery of currently undetectable biomolecules and the understanding of molecular recognition between biomolecules, and the development of novel disease markers and drugs [88]. Also the biomolecular nanoarrays have been applied to biosensors or biochips with high-throughput screening ability [130, 131]. Nanoarray of probe DNA enables the development of DNA sensors with high-throughput screening ability due to faster speed and massive parallelization [83].

Practically nanoarrays of antibodies with well-defined feature size and spacing enable highly sensitive and selective immunoassays to detect macromolecules in complex solutions. Lee *et al.* [130] showed that human immunodeficiency virus type 1 (HIV-1) in blood samples could be detected in DPN-generated nanoarrays of antibody, where HIV-1 antigen was bound to an antibody array and then the bound antigen was hybridized to gold antibody-functionalized nanoparticle probes for signal enhancement. The detection limit of nanoarray-based biosensing was more than 1,000-fold better than that of conventional enzyme-linked immunosorbent assay (ELISA)-based biosensing. Furthermore multi-biomolecular functional devices with multi-protein nanoarrays such as antibodies and enzymes could be fabricated via direct-write DPN. Chemically modified AFM tips enabled two-component nanoarrays of native proteins and were thus appropriate in the construction of novel biosensors and biochips with multi-functionality [131].

Nanomaterials have been effective in the development of highly sensitive devices for application in optics and electronics due to their low dimensionality and quantum confinement effect which are different from bulk structures or thin films [115, 132, 133]. Nanowires and nanotubes enable high sensitive detection owing to the change in charged carriers, which is caused by the binding of a charged biomolecule at the surface. The nanotubes and nanowires configured as field-effect transistors (FETs) have been used for the direct electrical detection of biomolecules due to the conductance change in FETs according to the binding of charged target biomolecules to their receptor attached to the surface [134].

Recently label-free biosensors with high sensitivity have been fabricated by using nanotube and nanowire FETs [135, 136]. The use of nanowire or carbon nanotube (CNT) transistors as an active transducer in the fabrication of biosensors enabled the real-time detection of single viruses, small molecules, and proteins [136, 137]. Semiconducting nanowires enabled the highly sensitive, specific label-free detection of antibodies as well as real-time monitoring of the cellular immune response, and the detection limit was below 100 femtomolar concentrations [136]. Furthermore nanoscaled fiber-type biosensors can directly monitor biomarkers inside a single cell [138].

### 5.2. Detection of molecular binding at the nanoscale

The molecular binding at the nanoscale can be divided into four major mechanisms- DNA hybridization, antigen-antibody, enzyme reaction, and specific molecular recognitions (e.g. biotin-avidin, protein A/G-antibody, and aptamer-receptor).

#### 5.2.1. DNA hybridization

DNA hybridization, which is caused by the complementary interaction between probe DNA and sample DNA, can be monitored through fluorescent detection using fluorescence-tagged probe DNA, or colorimetric detection using nanoparticle-conjugation, or electrochemical detection using indicators, as well as multiplication of sample DNAs by using polymerase chain reaction (PCR) (Table 4).

**Figure 13.**
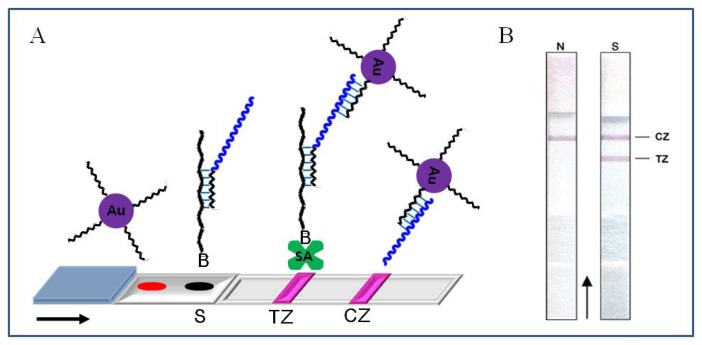
(A) Schematic illustration of the principle of the nanoparticle-based DNA biosensor for visual detection of GMO. The first red line is formed in the test zone (TZ), as the immobilized streptavidin captures the hybrids. The second red line is developed on the control zone (CZ), when the excess of conjugated gold nanoparticles hybridize with immobilized oligo(dA) probe. Au, conjugated gold nanoparticles; B, biotin; SA, streptavidin. (B) The hybridization assay of amplified lectin DNA by using the biosensor. PCR negative (N) and positive samples (S) are shown. TZ, test zone and CZ, control zone. Reproduced with permission of Elsevier [141].

Recently Kalogianni *et al.* [141] developed a nanoparticle-based DNA biosensor for visual detection of genetically modified organisms (GMO). The target sequence in GMO samples was simultaneously amplified and biotinylated by polymerase chain reaction (PCR), and then biotinylated PCR product was immobilized to streptoavidins on the test zone (TZ) of the sensor via biotin-avidin interaction. The biotinylated sample DNA was hybridized with complementary oligonucleotide probes containing an oligo(dA) tail. Oligo(dT)-conjugated gold nanoparticles were coupled with the target DNA through poly(dA/dT) hybridization. The excess of the oligo(dT)-conjugated gold nanoparticles was hybridized with immobilized oligo(dA) probe on control zone (CZ). The positive samples containing target DNA sequence represent the red band in color on both TZ and CZ, while the negative samples without target sequence show the color only on CZ (Figure 13). The red color originates from gold nanoparticles because they absorb the optical wavelength of around 520 nm due to surface plasmon resonance [141].

#### 5.2.2. Antigen-antibody

The molecular binding of antigen-antibody, which is caused by the high specificity between an antibody and an antigen, can be detected by optical methods such as fluorescent labeling and localized surface plasmon, or electrochemical method (Table 5). Recently Lin *et al.* fabricated an electrochemical biosensor using antibody-conjugated CdSe@ZnS quantum dot (QD) as a signal-amplifier vehicle for rapid and sensitive detection of specific antigen in human serum. The nanoparticle-label/immunochromatographic electrochemical biosensor (IEB) represented high sensitivity with the detection limit of 0.02 ng/mL prostate-specific antigen (PSA) which is the most reliable tumor marker to detect prostate cancer [142].

#### 5.2.3. Enzyme reaction

Enzyme reaction occurs by molecular binding of reactant to an active site of the enzyme. The catalytic activity of enzymes on surfaces modified at the nanoscale can be detected either directly or in conjugation with nanocomposites through electrochemical or optical methods (Table 6). Especially the reactions of redox enzymes like flavoenzyme, GOx, lactate oxidase and peroxidase have mainly detected the change in electrochemical signal due to the electrical communication between the biocatalysts and electrodes. However, the electron transfer rate constants between electrodes and enzymes are too low compared to those between enzymes and O_2_, a natural electron acceptor. The enhancement of electron transfer between redox enzymes and electrodes has been achieved with the help of conductive or semiconductive nanoparticles [117, 128].

**Figure 14.**
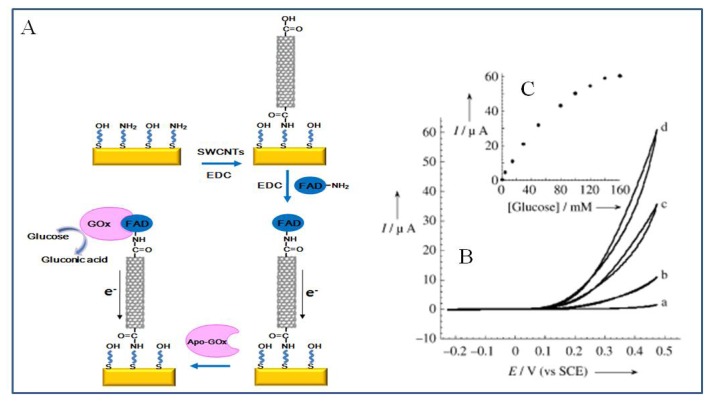
(A) Assembly of the SWCNT electrically contacted GOx electrode. (B) Cyclic voltammograms corresponding to the electrocatalyzed oxidation of different concentrations of glucose by the GOx reconstituted on the 25 nm long FAD-functionalized CNTs assembly: a) 0, b) 20, c) 60, d) 160 mM glucose. (C) Calibration curve corresponding to the amperometric responses of the reconstituted GOx/CNTs electrode in the presence of different concentrations of glucose. Reproduced with permission of I. Willner [124].

Figure 14 shows the electrochemical detection of the catalytic activity of aligned GOx on the edge of carbon nanotubes that are linked to gold electrodes. Carboxyl groups at the CNT ends were covalently attached to amino groups on the gold electrode surface and then FADs were sequentially immobilized on the opposite ends of CNT by EDC-mediated chemical reaction. Apo-GOx was specifically bound with FAD cofactor, thereby creating GOx holoenzyme with catalytic activity. Immobilized GOx converts glucose into gluconic acid and simultaneously produces electrons that are transferred to the gold electrode through the CNT. The electrocatalytic anodic current increases in proportion to the increase of glucose concentration [124].

#### 5.2.4. Specific molecular recognitions

Specific molecular recognitions such as avidin-biotin, protein A/G-antibody, and aptamer-receptor can be detected by optical or electrochemical methods (Table 7). Recently aptamers have been introduced as molecular recognition elements for the fabrication of biosensors [118, 144]. Aptamers are natural or artificial oligonucleotides that can bind to cellular or viral proteins with high selectivity, specificity and affinity. The aptamers are relatively inexpensive and stable compared to antibodies. The specific binding of an analyte to an aptamer on an electrode leads to a change in signal, thus being detected electrochemically [137, 144].

So *et al.* [137] fabricated an electrochemical aptamer biosensor on a SWCNT-field effect transistor (SWNT-FET) for the detection of thrombin. The SWCNT surfaces can easily be functionalized by aptamers via covalent bonding.

**Figure 15.**
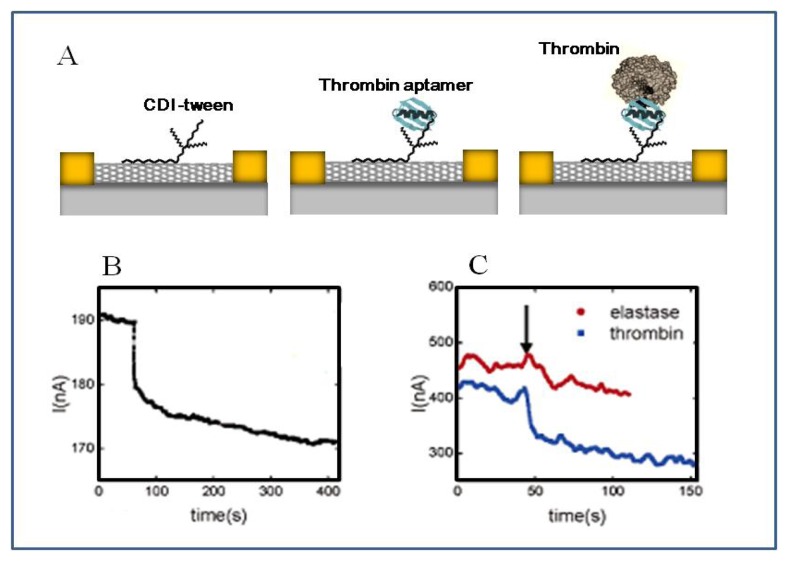
(A) Binding of thrombin on a SWCNT-FET-based aptamer sensor (B) A real-time conductance measurement obtained from the thrombin aptamer immobilized SWCNT-FET. (C) The selectivity of thrombin aptamer immobilized SWCNT-FET. Arrow indicates the point of adding protein solution. Reproduced with permission of ACS [137].

A 15-mer ssDNA aptamer that specifically binds to thrombin was immobilized on a SWNT-FET. Tween moiety of carbodiimidazole-activated Tween-20 (CDI-Tween) was bound to the side-wall of the carbon nanotube through hydrophobic interaction and then the thrombin aptamer was covalently attached by chemical reaction between the carbodiimidazole moiety on CDI-Tween surface and the amine group of the aptamer. While the addition of another protein (elastase) did not affect the conductance of the thrombin aptamer functionalized SWNT-FET, the binding of thrombin caused a sharp decrease in conductance, showing the selectivity of a biosensor using immobilized aptamer (Figure 15).

## 6. Future directions

Significant progress has been made in the development of methods for building nanostructures and nanostructured materials out of biological components such as oligonucleotides, proteins and enzymes. In this review, the relevance of surface chemistry based on molecular recognition and specific interactions has been reviewed, especially within the context of fabrication of novel biosensor. It is foreseeable that the role of self-assembly based on molecular recognition and specific interactions for the fabrication of highly defined surface architecture will further increase with the development of micro- and nanometer scale physical manipulation techniques, such that single molecules would be not only deposited onto a spatially defined location, but also individually addressable. We truly believe that the combination of the current available top-down fabrication techniques with bottom-up approaches may guide the research of bio-nanotechnology into a new era.

## Figures and Tables

**Table 1.** Major functional tailgroups for surface functionalization.

**Functional groups**	**Surface property**	**Interaction or reaction with biomolecules**
**R-(CH_2_)n-NH_3_^+^**	(+) Charge	- Charge-charge interaction - EDC-mediated chemical bonding with carboxyl group (R-COOH) of biomolecules - Glutaraldehyde-mediated chemical bonding with amine group (R-NH_2_) of biomolecules
**R-(CH_2_)n-COO^-^**	(-) Charge	- Charge-charge interaction - EDC-mediated chemical bonding with amine group of biomolecules
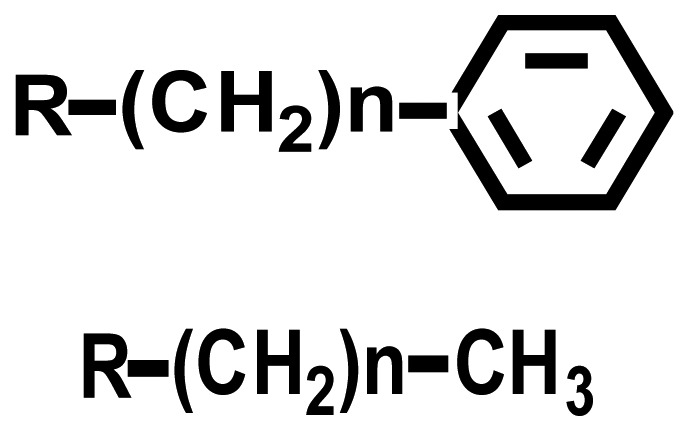	Hydrophobic	- Hydrophobic interaction
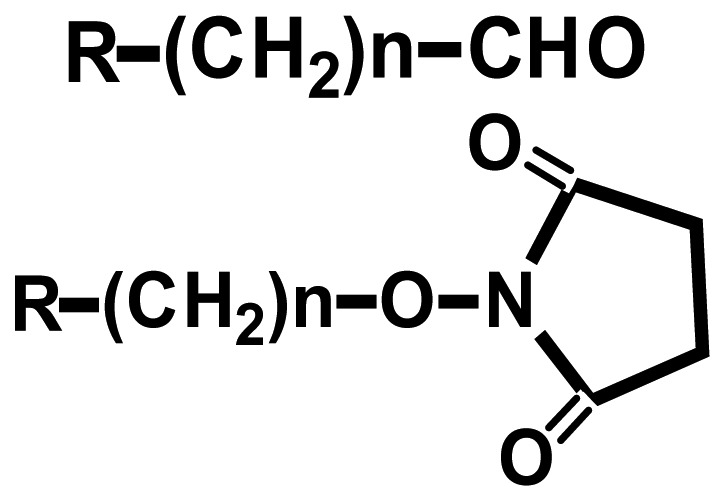	Aldehyde NHS	- Chemical bonding with amine group of biomolecules
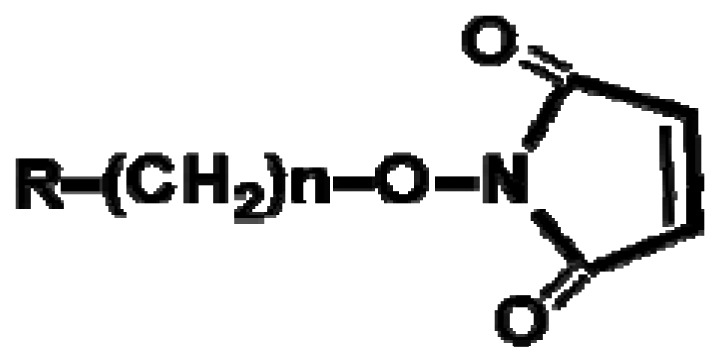	Maleimide	- Chemical bonding with sulfhydryl group (R-SH) of biomolecules
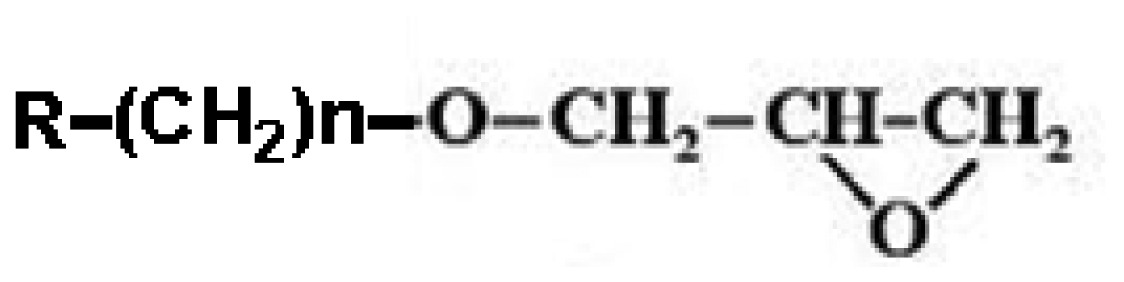	Epoxy	- Chemical bonding with hydroxyl (R-OH), amine and sulfhydryl groups of biomolecules
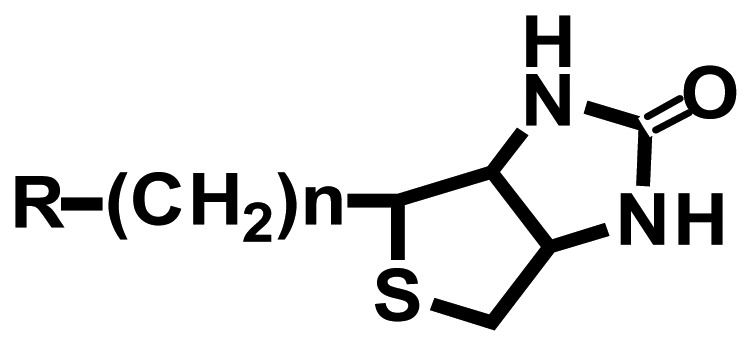	Biotin	- Specific interaction with avidin or streptavidin
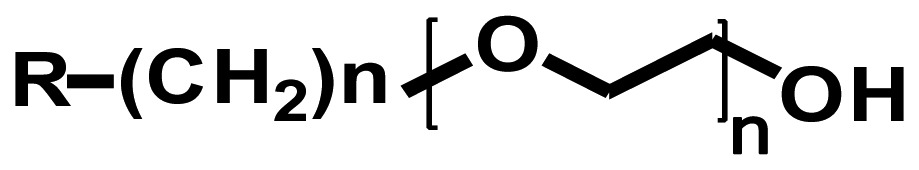	Ethylene glycol	- Preventing the non-specific adsorption of biomolecules

**Table 2.** Immobilization routes of functional biomolecules on functionalized surfaces.

**Routes**	**Advantages**	**Drawbacks**	**Bond energy (kcal/mol)**
Electrostatic interaction	Simple, Fast, Reversible, Direct method (no linker molecules), Retention of the natural 3D structure	Desorption by change of ionic strength or pH, Random orientation	< 5 (individually weak but collectively strong)
Hydrophobic interaction	Simple, Fast, Direct method (no linker molecules), Suitable to lipophilic biomolecules	Desorption by detergent, Random orientation, Denaturation of soluble biomolecules	
Covalent bonding	Good stability, High binding strength Use during long term	Random orientation, Use of linker molecules, Slow, Irreversible	∼100
Specific interaction	Improved orientation, High specificity and functionality, Well-controlled, Reversible	Use of biocompatible linker molecules, Expensive, Slow	–

**Table 3.** Nanoscale patterning methods using biomolecules [84, 85, 88].

**Technique**	**Advantages**	**Limitations**	**Highest Resolution**
Electron beam lithography (EBL)	Maskless, Stampless, High-resolution, Arbitrary patterning with different shapes and sizes	Slow (serial process), Complicated, Expensive (Requiring equipment, clean room and vacuum condition), Small area patterning	∼30 nm [89]
Nanocontact printing (NCP)	Simple (direct patterning), Parallel, Cheap, Fast process, Large area patterning	Preparing nanoscale stamp with high feature density, Mechanical stability of stamp, Diffusion of SAM inks	∼70 nm [90]
Nanoimprint lithography (NIL)	Large area patterning with a high-throughput and low-cost, Parallel	Stress and wear of mold, Use of polymer, Slow (molding, demolding, and etching process)	∼75 nm [91]
Nanografting/ Nanoshaving	High-resolution, Ambient, Quick change of fabricated patterns	Small area patterning	∼10 nm [92]
Dip-pen nanolithography (DPN)	High-resolution, Ambient, Variety of inks usable, Parallelization possible	Slow (serial process), Small area patterning	∼30 nm [93]

**Table 4.** Detection of DNA hybridization at the nanoscale.

**Nanostructures**	**Description**	**Detection**	**Ref.**
DPN-generated nanoarray	Two-sequence DNA array High-throughput screening	Epifluorescence	[113]
Gold nanoparticle + silver enhancement	Ultra-sensitive colorimetric biosensor	Absorbance at 630 nm	[139]
CNT + phalladium nanoparticle + indicator	Ultra-sensitive electrochemical biosensor	Differential pulse voltammetry	[140]
Gold nanoparticle	Visual detection of genetically modified organisms (GMO)	Colored band	[141]

**Table 5.** Detection of antibody-antigen interaction at the nanoscale.

**Nanostructures**	**Description**	**Detection**	**Ref.**
DPN-generated nanoarray + gold nanoparticle	Detection of human immunodeficiency virus type 1 (HIV-1)	RT (Reverse transcriptase)-PCR-based assay	[130]
CNT + poly(ethylene vinylacetate) (EVA)	Use of ruthenium(II) tris(2,2′-bipyridine)-conjugated antibody	Electrochemiluminescen ce (ECL)based assay	[139]
Surface modified gold- capped nanoparticle layer	Optical biosensor with 10 ng/ml of detection limit	Localized surface plasmon resonance	[127]
CdSe@ZnS QD as a signal-amplifier vehicle	Rapid, sensitive detection of PSA in human serum	Electrochemical	[142]

**Table 6.** Detection of enzyme reaction at the nanoscale.

**Nanostructures**	**Enzyme**	**Description**	**Detection**	**Ref.**
Gold nanoparticle	GOx	Use of apo-GOx-FAD cofactor specific interaction	Electrochemical	[128]
ZnO nanoparticle	Micro- peroxidase	Ultra-sensitive colorimetric biosensor	Electrochemical	[117]
SWNT forests	HRP	Ultra-sensitive electrochemical biosensor	Electrochemical	[121]
CdTe QD + CNT	GOx	High sensitivity	Electrochemical	[143]
CNT	GOx	Use of apo-GOx-FAD cofactor specific interaction	Electrochemical	[124]

**Table 7.** Detection of specific molecular recognitions at the nanoscale.

**Nanostructures**	**Molecular recognition**	**Detection**	**Ref.**
NIL-generated nanopattern	Biotin-streptavidin	Epifluorescence	[91]
EBL-generated nanopattern	Biotin-streptavidin Streptavidin-biotin-GFP	Epifluorescence	[94]
Gold nanotube	Biotin-streptavidin Streptavidin-biotin-protein G Protein G-antibody Antibody-antigen	Electrochemical	[122]
CNT	Biotin-streptavidin Protein A-antibody	Electrochemical	[125]
CNT-FET	Aptamer-Immunoglobulin E	Electrochemical	[118]
SWCNT-FET	Aptamer-thrombin	Electrochemical	[137]
